# Range extension of a boreal amphipod *Gammarus oceanicus* in the warming Arctic

**DOI:** 10.1002/ece3.4281

**Published:** 2018-07-09

**Authors:** Jan Marcin Węsławski, Katarzyna Dragańska‐Deja, Joanna Legeżyńska, Waldemar Walczowski

**Affiliations:** ^1^ Institute of Oceanology Polish Academy of Sciences Sopot Poland

**Keywords:** Amphipoda, Arctic, distribution shift, Gammarus, global change, intertidal, Svalbard

## Abstract

The recent (2008–2016) occurrence of a boreal intertidal amphipod *Gammarus oceanicus* along the Spitsbergen coast is compared with corresponding data from 1980 to 1994. We aimed to compare the pace of environmental changes in the area (ice retreat, temperature increase) with distribution change of *G. oceanicus*. Material for the study was collected from intertidal, at low water level from over 100 locations on Spitsbergen, the main island of Svalbard archipelago (expanding from 76 to 80°N). The west coast of the island has been exposed to a steady increase in sea surface and air temperature (2°C in 20 years), as well as a significant decrease in fast ice duration (from over 5 months to less than 1 per year). A total length of more than 3,600 km of the island's coastline has been recently impacted by warming. Of the two sibling *Gammarus* species that dwell in the Spitsbergen littoral, *G. setosus*, the local cold water species remains generally where it was observed about 20–30 years ago. By contrast, boreal *G. oceanicus* has expanded its distribution range by over 1,300 km along the west and north coasts of Spitsbergen and gained dominating position on the number of sites, where it was previously just an occasional species.

## 1 INTRODUCTION

The unique position of the European Arctic on the map of changing climate is not disputed; the rate of warming in this region has never been so fast as in the last 20 years and is not likely to slow down in the coming years (ACIA 2005, IPCC 2014). Climate warming in the Arctic is manifested by several interdependent physical phenomena: a rise of air temperature, an increase in the volume of warm Atlantic waters transported northward by the North Atlantic current, a loss of fast ice on the coast (duration, area and thickness) and pack ice retreat (duration, area, thickness). Most probably, these phenomena are responsible for the observed heightened wave erosion of the shores and coastal water turbidity. Marine invertebrates are often regarded as good indicators of environmental conditions (Blacker, 1957; Ikko & Lyubina, 2010; Węsławski, 1994). Along with the change of physical conditions in the Svalbard archipelago, changes in the local fauna have been recorded. The most notable were the discovery of the thermophilic bivalve *Mytilus edulis* population in Isfjorden (Berge, Johnsen, Nilsen, Gulliksen, & Slagstad, 2005) and the appearance of Atlantic cod, mackerel, and pipefish along Spitsbergen (Fleischer, Schaber, & Piepenburg, 2007; Hopkins, 2002). Furthermore, thirty years of observations in two Spitsbergen fjords documented extensive structural changes in the rocky‐bottom communities with the abrupt increase in macroalgal cover and the simultaneous reorganization in the invertebrate assemblage as a consequence of temperature increase and fast ice disappearance (Beuchel & Gulliksen, 2008; Beuchel, Gulliksen, & Carroll, 2006; Kortsch et al., 2012; Węsławski et al., 2011). Also, the northward advance of snow and king crabs (Jørgensen, Løkkeborg, Fernö, & Hufthammer, 2007; https://www.unis.no/snow-crab-arrived/) and ornithological records of northward shifts of the nesting areas of gannets, great skuas, ravens, swans, and cormorants (Dr H. Strøm, Norwegian Polar Institute, pers. comm.) confirm an impact of warming on Arctic fauna.

Intertidal fauna dwelling in polar regions experiences extreme fluctuations of salinity and temperature and devastating disturbance caused by scouring of land fast ice on a regular basis. Nevertheless, intertidal organisms are abundant and common along the Spitsbergen coast (Węsławski, Wiktor, Zajączkowski, & Swerpel, 1993). Among them, two species of the amphipod genus *Gammarus* dominate intertidal fauna at localities covered with loose stones and rocks that protect them during low tides. The cold water, Arctic species *Gammarus setosus* is widely spread in the Arctic including various localities around Spitsbergen (Gurjanova, 1951; Stephensen, 1935; Tzvetkova, 1975; Węsławski, 1994). Its sibling species, *Gammarus oceanicus*, is supposedly of Mediterranean origin, but has a boreal distribution along the northern coasts of Europe and North America (Jażdżewski, 1970; Lincoln, 1979; Tzvetkova, 1975). Due to morphological and ecological similarities prior to the original description of *G. oceanicus* (Segerstråle, 1948), both species were often confused. The earliest reliable report of *G. oceanicus* occurrence on Spitsbergen comes from Hornsund (77°N), where it was found in 1957 and described by a trained amphipod taxonomist (Micherdziński, 1959). Both species are free‐moving, relatively large (up to 35 mm long) perennial (3–4 year life span) crustaceans with a direct development, occupying basically the same type of habitat and making use of the same food sources (Steele & Steele, 1970, 1972, 1974; Tzvetkova, 1975; Węsławski, 1994; Węsławski & Legeżyńska, 2002). *Gammarus* individuals locally and populations globally are very tolerant to temperature and salinity fluctuations, being found at salinities between 0 and 35 and in temperatures between ‐1.5 and 10°C (even as high as 25°C in the case of *G. oceanicus*) (Segerstråle, 1948; Tzvetkova, 1975; Węsławski, 1994). According to Węsławski (1994), *G. setosus* and *G. oceanicus* co‐occur along the Spitsbergen coast, with the former species preferring the inner fjord basins and the east coast of the island and the latter one being restricted to the west coast influenced by the warm west Spitsbergen current. The fact that the sibling *Gammarus* species are indicators of environmental conditions (Ikko & Lyubina, 2010) was a reason for repeating the littoral sampling 20 years after the first such collection (Węsławski, 1994). Fast ice disappearance and increase of Atlantic waters’ inflow, followed by air and water temperature rise, are factors considered as beneficial for *G. oceanicus* expansion. The obvious working hypothesis was that the warm‐water species, *G. oceanicus*, would expand its range in the wake of observed warming.

### 1.1 Study area

**Figure 1:**
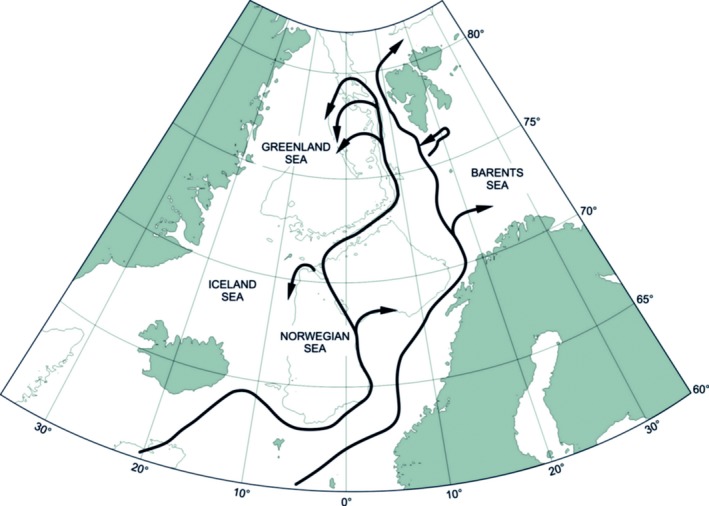
The two main branches of Atlantic water that may carry boreal organisms from Europe to Spitsbergen (Walczowski, Piechura, Goszczko, & Wieczorek, 2012, modified)

Spitsbergen, the main island of the Svalbard archipelago, is exposed to different water masses. Its west coast is directly influenced by the west Spitsbergen current, the main inflow of warm Atlantic waters to the Arctic, while on the east cold local waters of Arctic origin dominate (Figure 1). The surface sea currents show the ways of Atlantic water transport from Europe to Spitsbergen (Figure 1). Pack ice from the Barents Sea is common for part of the year along the east coast of Spitsbergen, but rare on the west coast (mostly short episodes in summers). Fast ice is common in all sheltered and landlocked fjord basins and sheltered bays around the island. In the colder, eastern area, it occurs also along the open coast (http://www.meteo.no.ice). Spitsbergen intertidal zone is diverse with numerous stony beaches and rocky promontories (Węsławski, Wiktor, et al., 1993).

## 2 MATERIAL AND METHODS

### 2.1 Sampling

**Figure 2:**
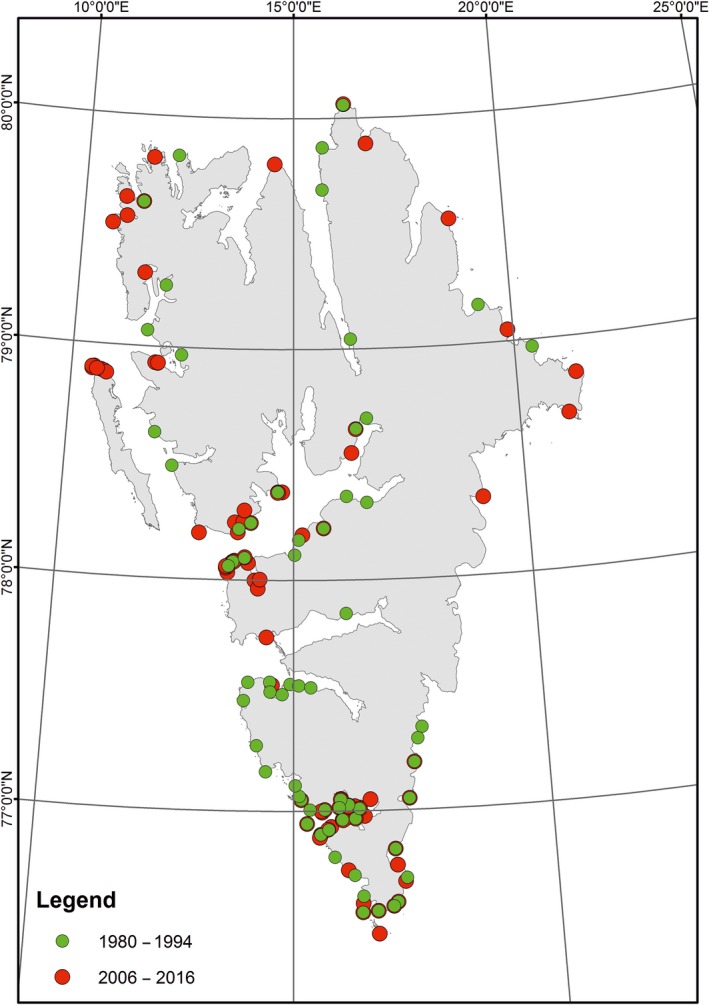
The intertidal collection of *Gammarus* species along Spitsbergen shores in the years 1980–1993 and 2008–2016

Intertidal sampling of amphipods took place as part of the Svalbard Intertidal Project (http://water.iopan.gda.pl/projects/SIP/index.html) during six summers 2008–2010 and 2014–2016 (Figure 2). The most coherent was the samples’ collection in 2015, when the circumnavigation of Spitsbergen was completed in one season. Archival material on *Gammarus* occurrence from 1980 to 1994 (Węsławski, 1994; Węsławski, Kwaśniewski, Wiktor, & Zajączkowski, 1993; Węsławski, Wiktor, Zajączkowski, Futsaeter, & Moe, 1997; Węsławski, Wiktor, et al., 1993) was used for comparison. Sixty‐four stations were sampled during the archival survey and 151 between 2008 and 2016 (see the Supporting Information Appendix S1). Amphipods were collected with a small hand net, at low tide, below loose stones at the low water mark. Between 20 and 100 specimens from each site (~1 m^2^) were collected and preserved in ethanol. Only specimens longer than 5 mm with well‐developed diagnostic features were identified. The geographic position of each site was recorded, photographs taken, and the air and sea temperatures noted; all these data can be found on the above mentioned Web site. Hydrographic data were collected along the west Spitsbergen shelf and in three fjords (Hornsund, Isfjorden, and Kongsfjorden) during routine summer cruises of r/v Oceania, in July/August each year since 1988 (Walczowski, 2014; Walczowski & Piechura, 2011; Walczowski, Piechura, Osiński, & Wieczorek, 2005). Data on fast ice extension and duration were taken from the Norwegian Meteorological Service—http://www.meteo.no/ice, where satellite‐based information is presented for each month starting from 2001. Estimates of fast ice in 1980–1990 were based on the information taken from several published studies (Smith & Lydersen, 1991; Węsławski, Kwaśniewski, 1993; Węsławski et al., 1997), as well as data from own unpublished reports and observations.

### 2.2 GIS model

Assumptions to the GIS modeling were as follows:

1. Intertidal *Gammarus* needs loose stones in moderately exposed areas, as the key shelter during low tide. Such habitat is common and widespread along entire Spitsbergen coast, and genus *Gammarus* occurs in all proper sites (Węsławski, 1994).
2. This habitat offers a finite space, and the density of *Gammarus* in a particular shelter cannot increase ad infinitum.
3. As the density of gammarids under the stones was very similar in different sites (Węsławski, 1994), the assumption is that available habitat (space) has been occupied by the species that colonized the coastline first (cold water *G. setosus*).
4. Hence, the species that comes next needs to compete for space (save shelter under the stone). The suboptimal habitat like bushy algae may offer additional food that is of no critical importance for the omnivorous *Gammarus*.

The comparison between old and recent data was organized on a grid of 5 × 5 km squares superimposed on the Spitsbergen coast line, the same grid that was used in the oil spill vulnerability survey (Węsławski et al., 1997). The center of each square was given a geographic position, and three categories of fast ice (<1 month per year, 1–5 months per year, and more than 5 months per year) were used to describe the square. In order to illustrate the occurrence of *G. oceanicus* along the Spitsbergen coast, data from point stations were interpolated (Spline with Barriers tool; ArcMap software; Supporting Information Appendix S1). The resulting rasters were set to resolutions of 5 × 5 km (see Figure 5) and 10 × 10 km (occurrence of *Gammarus*). Raster cell values were reclassified into three sets—with *G. oceanicus* shares of 51%–100%, 1%–50%, and 0%, the last set implying the exclusive presence of *G. setosus*. Stations where single specimens were collected were omitted from GIS analysis.

## 3 RESULTS

**Figure 3:**
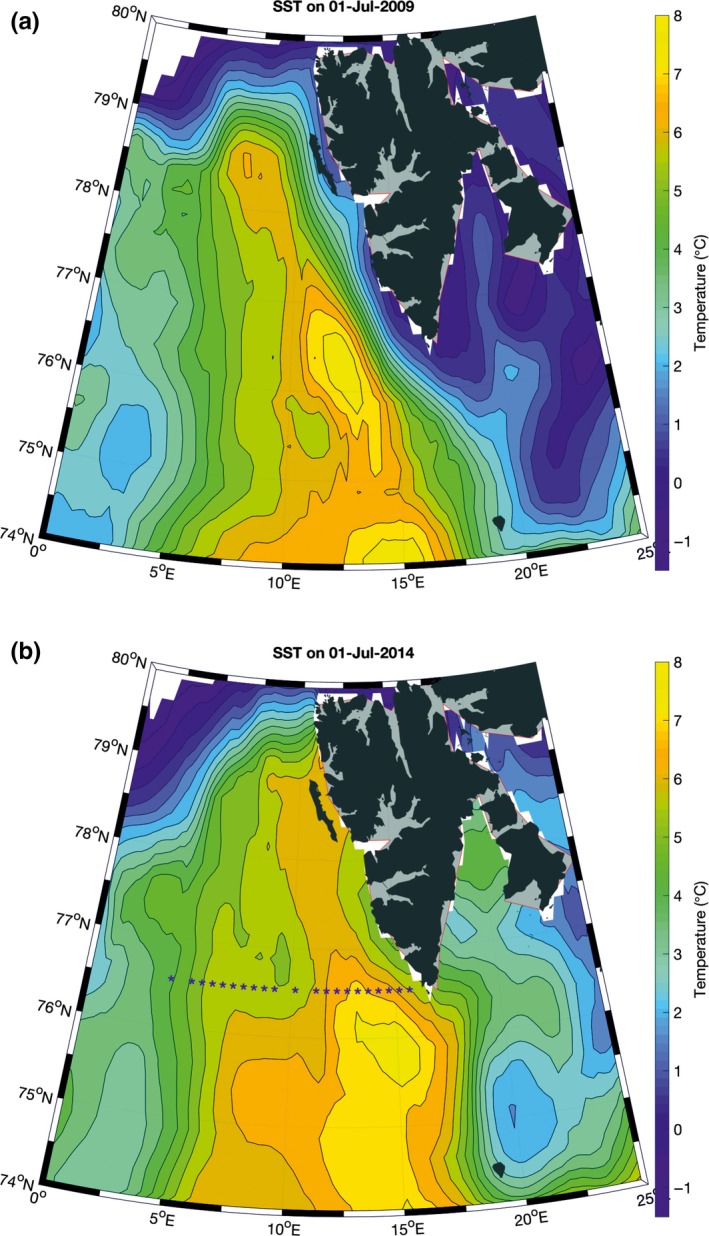
Sea surface temperature distribution in July 2009 (a, showing the warm water of Atlantic origin separated from the coast [a]) and in July 2014, when warm water expanded toward the coast and fjords. (b) Based on the optimum interpolation sea surface temperature (OISST) data (Reynolds et al., 2007)

**Figure 4:**
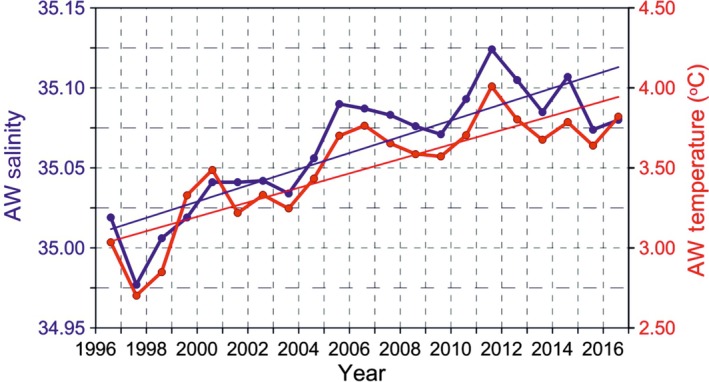
Temperature and salinity changes in Atlantic Waters at transect off the Spitsbergen coast (76°30′, indicated in Figure 3b). IOPAS data, Atlantic water defined within the density range 27.7–27.97. The surface advective layer was excluded

**Figure 5:**
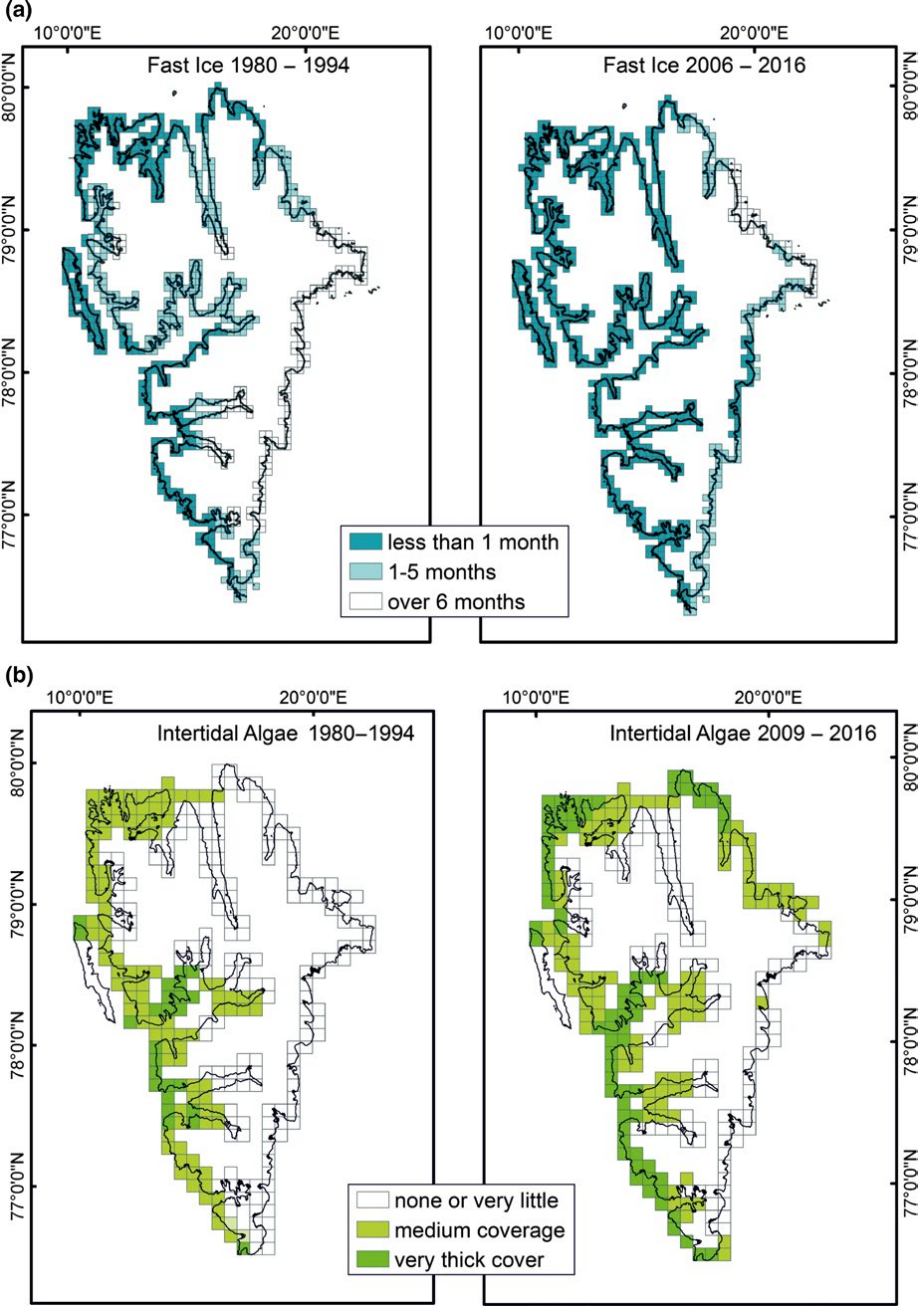
Fast ice duration (a) and intertidal algal coverage (b) in the years 1980–1993 and 2008–2016 on Spitsbergen island; data from http://www.meteo.no/ice and own data

The water mass properties along the Spitsbergen coast varied year on year and changed with respect to both the northernmost position and temperature of the Atlantic water tongue and its proximity to the fjord entrance (Figure 3). The local cold water of the Sørkapp current from east Spitsbergen may form a barrier between the warm Atlantic waters and the Spitsbergen coast (as observed in 2009, Figure 3). When strong southeasterly winds blow, Ekman transport may break this barrier, enabling warm water to flow in over the shelf and enter the west Spitsbergen fjords (as observed in 2014, Figure 3). Based on the long‐term hydrological and meteorological observations (Walczowski, 2014; Figure 4), the years 1997, 2001, 2003, 2010, and 2011 were regarded as “cold” and 2000, 2002, 2006, 2007, and 2014 as “warm,” which corresponds with the NOA winter index (National Centre 2016). The presence of freshwater along the coast was the most stable factor, while pack ice and fast ice were the most changeable (Supporting Information Appendix S1). The length of the coastline with diminished fast ice cover differed between cold and warm years by more than 3,600 km (Figure 5).

**Figure 6:**
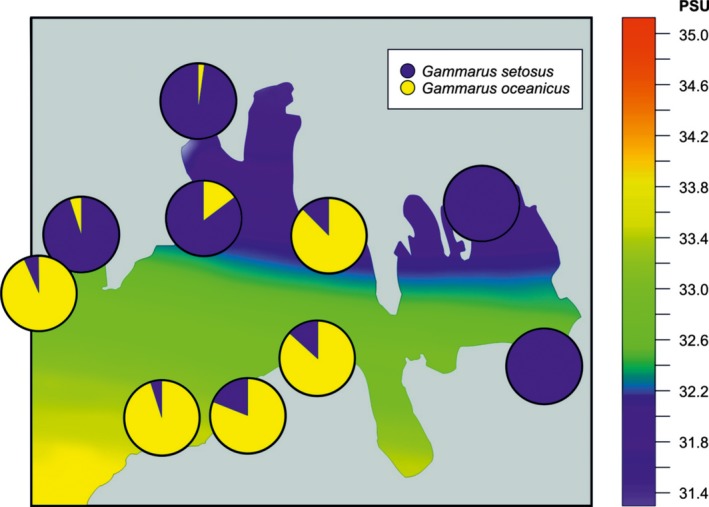
Inflow of shelf waters along the southern shore and outflow of fjord water along the northern shore of a west Spitsbergen fjord (here Hornsund, July 2008) and the occurrence of *Gammarus oceanicus*

The circulation in all the west Spitsbergen fjords generally follows the pattern of shelf waters flowing in along the south coast and out along the north coast: This is reflected by the surface salinity distribution. This type of water dynamics is driven by a cyclonic circulation and the prevailing easterly winds that drive water along the north coast of the fjord (Ekman transport). The local distribution of *G. oceanicus* within the fjord corresponded with the incoming water (Figure 6).

Investigated coastline was estimated to be 6,600 km and divided into 302 10 × 10 km squares, of which gammarids were collected from 49 squares in the 1980s (16% of all) and from 46 squares in 2008–2016 (15% of all). The combined effect of the reduction of fast ice presence, summer temperature of coastal water above 3°C, and presence of intertidal algae was considered as favorable habitat settings for *G. oceanicus*. Such conditions were found along 2152 km of Spitsbergen coastline in the 1980s and expanded by ca. 30% during last 20–30 years (Table 1). The fast ice reduction alone occurred over much larger area (up to 3,000 km, Figure 5), but ice conditions were very variable from year to year. Both in the previous and in recent surveys, *G. oceanicus* occurred in slightly wider area than the most favorable habitat (35% of the coastline occupied versus 33% coastline regarded as favorable in the 1980s and 55 versus 44% in recent collection—Table 1). Over the last three decades, *G. oceanicus* extended its geographic distribution along the Spitsbergen coast by over 1300 km (from 2,335 km in the 1980s to the present occurrence on 3,677 km, Table 1). At the same time, area in which the species dominated over an Arctic species *G. setosus* increased from 328 km to 1,833 km (Table 1). There were no sites where *G. setosus* was not observed in the 1980s, but appeared in recent samples (this might have suggested a range extension of this species).

**Table 1:** Length of the coast with *Gammarus oceanicus* occurrence in the 1980s and in 2008–2016

	1980–1993 (km)	1980–1993 (%)	2008–2016 (km)	2008–2016 (%)
Favorable habitat for *Gammarus oceanicus*	2,152	33	2,873	43
*Gammarus oceanicus* presence	2,335	35	3,677	56
*Gammarus oceanicus* domination (51%–100%)	328	5	1,833	28

**Figure 7:**
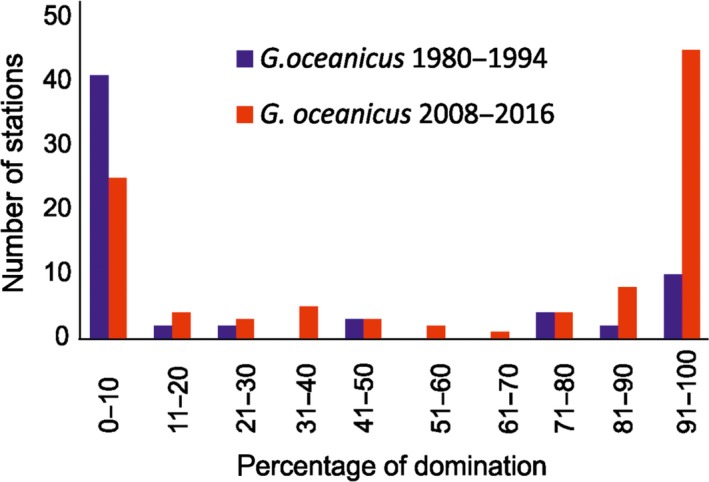
Percentage share of *Gammarus oceanicus* in the total numbers of collected *Gammarus* individuals in old and recent collection

**Figure 8:**
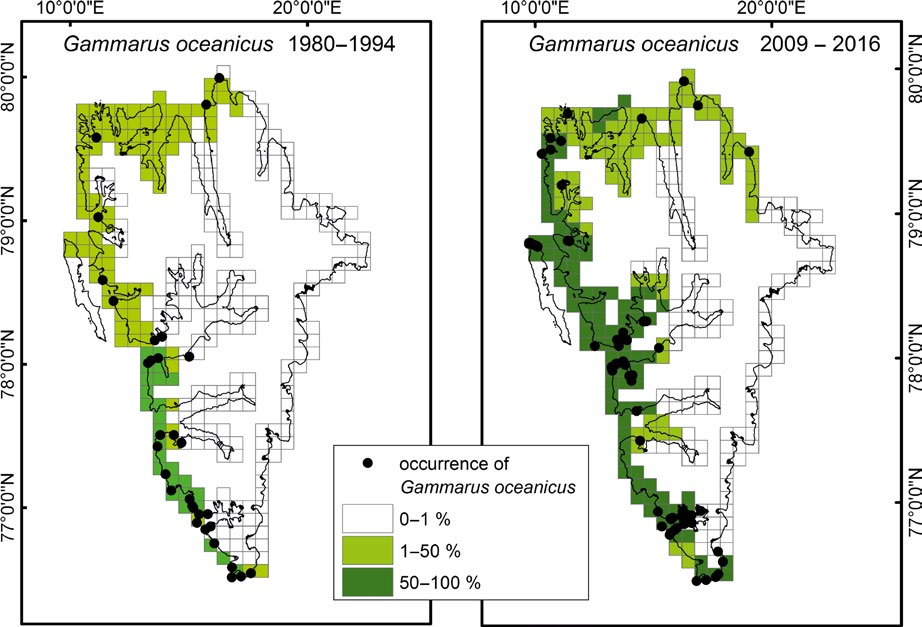
Occurrence of *Gammarus oceanicus* and its share in the total numbers of *Gammarus* spp. collected along the coast of Spitsbergen in the years 1980–1994 and 2008–2016

It appears that the recent rise of *G. oceanicus* population is mainly achieved through the density increase in already occupied areas (Figures 7 and 8). In recent years, the species reached a dominant position along 1,505 km (with assumed share 51%–100% of two species studied) and newly colonized (with assumed average share 1%–25%) ca. 1,300 km of the Spitsbergen coast. Figure 7 illustrates this, as the highest number of stations (52 stations) visited recently shows the very high dominance of *G. oceanicus* (this suggests that species was present in old collection and recently increased its share on the expense of local species). The stations, where *G. oceanicus* was found as a newcomer (percentage share 1%–25%), are not that numerous (25). This shows that population increase of *G. oceanicus* was attained by the increased competition on already occupied areas, while colonization of new grounds results in comparatively lower population increase.

## 4 DISCUSSION

The changes of the physical environment on the Svalbard coast are apparent: A detailed study on fast ice changes in Isfjorden and Hornsund (Gerland & Hall, 2006; Muckenhuber, Nilsen, Korosov, & Sandven, 2016) reports a drop from an average of 50 days with fast ice in early 2000 to 22 days in recent years. The inflow of Atlantic waters along the Spitsbergen coast tends to increase as a result of the general change in the North Atlantic circulation due to a long period of dominance of positive winter NAO index and is very strong (from 2 to 6, Sverdrup—Walczowski & Piechura, 2011). The intensity of warm‐water penetration from the shelf to the fjords is changeable, but it has been increasing steadily over the last 20 years (Cottier et al., 2010; Willis, Cottier, Kwaśniewski, Wold, & Falk‐Petersen, 2006). Following changes in the environment, changes in Arctic benthic communities have been documented, especially in shallow water habitats directly exposed to variability in temperature and ice conditions (Beuchel & Gulliksen, 2008; Beuchel et al., 2006; Kortsch et al., 2012; Węsławski, Wiktor, & Kotwicki, 2010).

The habitat preferences of the two *Gammarus* species dwelling in the Spitsbergen littoral are very similar (Steele & Steele, 1975; Tzvetkova, 1975; Węsławski, 1994). Nevertheless, there are good reasons to expect that *G. setosus* will perform better in a colder climate and *G. oceanicus* in warmer conditions (Steele & Steele, 1970, 1972, 1974). Rastric and Whiteley (2007)and Whiteley, Rastrick, Lunt, and Rock (2011) tested the physiological adaptations of *G. oceanicus* and *G. setosus* to Svalbard and reported that *G. oceanicus* exhibits a lower rate of protein synthesis at low temperatures compared to the local species, which confirms the better fitness of *G. setosus* to Arctic conditions. On the other hand, the current increase of water temperature would be more beneficial for thermophilic *G. oceanicus*. Ice loss would probably have similar, both positive (decrease of ice scouring intensity) and negative (higher exposure to predation risk, higher turbidity of water; Macneil et al., 1999, Cottier et al., 2010) consequences for the two species.

We found quite substantial over 1300 km advance in *G. oceanicus* range over the last 30 years (ca. 40 /year). *Gammarus* spp., like all peracarids, are brooders with dispersal potential assumed to be lower than that in taxa with a pelagic larval stage. While it is known that many crustacean species are highly mobile (Boudrias, 2013) therefore after an initial short‐distance dispersion of the juveniles, they may continue spreading over large distance through adulthood, and it is probably not the case of *Gammarus*. This genus is known to be more inclined to crawl or walk on the seabed than swim (Steele, 1988) and being a littoral dweller must hide under stones and among algae to avoid predators during low tides, which also limits its dispersal potential. Taking this into account, it appears that *G. oceanicus* dispersal must be accelerated by coastal sea currents. The direction of its advance generally tracks that of sea currents. The circulation models for the west Spitsbergen show that shelf waters enter fjords along their south coasts and leave along the north coast's (Cottier et al., 2010; Jakacki, Przyborska, Kosecki, Sundfjord, & Albertsen, 2017; Svendsen et al., 2002). Accordingly, *G. oceanicus* enters Hornsund and Isfjorden further along their south coast's than the north ones. Interestingly, the slow northeastward advance of *G. oceanicus* in Sørkappland (about 50 km) goes against the local Sørkapp current that carries cold waters from the Barents Sea in the northwestward direction along the Spitsbergen coast with velocities of up to 0.50 m/s (Walczowski, 2014; Walczowski et al., 2005).

It is not easy to compare the spread rate of *G. oceanicus* with those reported for other crustaceans as they differ in both species characteristics (e.g. size, mobility, behavior, and reproduction modes) and modes of dispersal. Sorte, Williams, and Carlton (2010) examined the published data on range extensions of marine native species (algae, plants, animals) in response to climate change and found that 75% of species had shifted poleward, the direction generally predicted by climate change. On average, range shifts occurred at rather slow pace of 19 km/year, but the rate can be much faster at high latitudes (28.0 ± 17.9 km/year). Maximal spread rates for Crustacea included in their data set (Cirripedia and crabs, both taxa with a planktonic stage) were estimated at 45–49 km/year. Little information has been published on the rate at which crustacean species expand their distribution in the Arctic. Jørgensen et al. (2007) studied the movement pattern of red king crab introduced to the Barents Sea and found that it is strongly connected with foraging strategy. The crabs were capable of moving at high speed (up to 270 m/hr) between food patches, but mostly remained stationary or moved slowly (<0.001 m/s). Therefore, their actual dispersal effectiveness is more likely limited by their behavior and lifestyle than by the potential speed of movement.

In general, local species that gradually colonize neighboring habitats spread slower than invasive species (Sorte et al., 2010). For example, a North American amphipod *Gammarus tigrinus* was first discovered in the Baltic Sea in 1975 (Schlei Fjord, Germany) and since then has spread along the south and eastern Baltic coasts, reaching the Gulf of Finland in 2003 (Jensen, 2010; estimated distance: ca. 3,680 km, pace of spreading: 131 km/year). Similarly, fast spread has been observed in the case of *Caprella mutica*, an amphipod native to the northeast Asia, which has become established in the North Sea, Scotland, and Ireland, in the Irish Sea and English Channel in <14 years (Cook et al., 2007), and has invaded several other regions, including North America, New Zealand, and South Africa over the last 50 years (Peters & Robinson, 2017). Rapid dispersal of these species, however, has been strongly enhanced by human activity, especially by ship traffic and aquaculture (Piscart, Maazouzi, & Marmonier, 2008).

In relatively pristine Svalbard area, dispersal rates can be mainly related to the species activity and hydrodynamic conditions. In addition to the range extension of the established population, dispersal of *G. oceanicus* may be enhanced by the advection of individuals from outside areas associated with increasing northward transport of Atlantic waters. The pathway of Atlantic waters indicates that *G. oceanicus* may be carried to Spitsbergen from various European locations where it is commonly noted, such as Iceland, Scotland, Norway, and the North Sea coasts (Bellan‐Santini & Costello, 2001; Hayward & Ryland, 1990; Krebes, Blank, & Bastrop, 2011; Lincoln, 1979).

Although modern GIS methods like climatic envelope modeling are promising tools for predicting species distribution changes (Drewnik, Węsławski, & Włodarska‐Kowalczuk, 2017; Goodenough & Hart, 2013), the case study presented here shows that physical conditions alone are not sufficient to explain changes in littoral community. It is probable that competition with well‐developed population of a native sibling species weakens the pace of *G. oceanicus* spreading along the Spitsbergen coast. On the other hand, growth of its population is mainly achieved by increase of its domination over *G. setosus* in the previously occupied areas. Usually, competitive superiority of the species is possible due to simultaneous occurrence of the several factors. Baltic invader, *G. tigrinus*, outcompetes native gammarid species by having a strong tolerance to variable environmental conditions, low habitat selectivity, early maturation, large brood size, short generation time, and aggressive behavior toward the coexisting species. It was also assumed that a presence of *G. tigrinus* increases the exposure of native species to fish predation (Kotta et al., 2013 and references herein). Further studies on interspecific interactions between two co‐occurring *Gammarus* species and an accurate recognition of the several aspects of their biology and ecology are needed to explain the success of *G. oceanicus* in the Spitsbergen littoral.

## CONFLICT OF INTEREST

None declared.

## AUTHOR CONTRIBUTIONS

JMW designed the study, collected and processed samples, and wrote the first version of the manuscript. KD‐D helped extensively with data analyses and provided several figures based on the GIS model. JL helped with the manuscript writing (ecological part) and editing. WW helped with the manuscript writing (hydrological part) and provided relevant figures. All authors critically revised and approved the final version of the manuscript submitted.
