# Supplementary material for: Range extension of a boreal amphipod Gammarus oceanicus in the warming Arctic
Source: Ecol Evol. 2018 Jul 9;8(15):7624–32. doi: 10.1002/ece3.4281 (PMC6106200; doi:10.1002/ece3.4281)
Supplement: Supplementary file 1 [file ECE3-8-7624-s001.docx]

**Range extension of a boreal amphipod *Gammarus oceanicus* in the warming Arctic.**

Węsławski J.M., Dragańska- Deja K., Legeżyńska J., Walczowski W. 2018

Paper submited to Ecology & Evolution

APPENDIX

Table 1. Localisation and habitat description of sampling sites at which *Gammarus oceanicus* and *Gammarus setosus* individuals were collected during the 1980-1993 and 2000-2016 sampling campaingns.

1. ICE: ice situation as was assessed from [www.meteo.no/ice](http://www.meteo.no/ice) and archival own data and codded according the following scale: 1- fast ice occurrence < 1 month, 2- fast ice occurrence 1-5 months, 3- fast ice occurrence > 6 month.

   2) ALGAE: algae cover on the intertidal was assessed from the visual survey of Svalbard intertidal project ([www.iopan.gda.pl/SIP](http://www.iopan.gda.pl/SIP)) and codded according the following scale: 1 - none (bare rocks or stones), 2- microbial film of green algae- microphytobenthos on hard surfaces, 3 – thick macroalgal cover (turfs of green and brown algae).

   3) WAVES: exposure to waves was assessed from the sea charts and observation of the coast ([www.iopan.gda.pl/SIP](http://www.iopan.gda.pl/SIP))  and codded according the following scale: 1- very exposed - open coast with direct oceanic waves, 2- partly sheltered fjords and large bays affected by local waves, 3- shetlered areas - innermost fjord basins, sheltered with islands with little exposure to local waves

   3) WATER MASSES: presence of Atlantic (ATL) and Arctic (ARC) waters was assessed from the hydrographic studies of present authors and literature

   4) FRESH: fresh water ccurrence was assessed from the hydrographic studies and observations of present autohrs and codded as: 0 – sites outside fresh water influence, 1- sites influenced by rivers, lakes or glacial outflow.

5) *GAMMARUS*  columns show the number of individuals identified to species level from the particular site

|  |  | Latitude | Longitude | ICE | ALGAE | WAVES | WATER MASSES | | FRESH | *G. oceanicus* | *G. setosus* |
| --- | --- | --- | --- | --- | --- | --- | --- | --- | --- | --- | --- |
| Collection | Site name |  |  |  |  |  | ATL | ARC |  |  |  |
|  |  |  |  |  |  |  |  |  |  |  |  |
| 1980-1993 | Schonrockfjellet | 77.3572 | 17.5375 | 3 | 1 | 2 | 0 | 1 | 0 | 0 | 15 |
| 1980-1993 | Brimoden | 77.3092 | 17.4475 | 3 | 1 | 2 | 0 | 1 | 0 | 0 | 15 |
| 1980-1993 | Crollbreen | 77.2062 | 17.3638 | 3 | 1 | 2 | 0 | 1 | 0 | 0 | 15 |
| 1980-1993 | Hambergbukta | 77.0505 | 17.2426 | 3 | 1 | 1 | 0 | 1 | 1 | 0 | 15 |
| 1980-1993 | Isbukta | 76.8326 | 16.9404 | 3 | 1 | 1 | 0 | 1 | 1 | 0 | 15 |
| 1980-1993 | Skolthuken | 76.7064 | 17.1436 | 2 | 1 | 2 | 0 | 1 | 0 | 0 | 15 |
| 1980-1993 | Kikutodden | 76.6027 | 16.9612 | 2 | 1 | 2 | 0 | 1 | 0 | 0 | 15 |
| 1980-1993 | Moloen | 76.5851 | 16.8797 | 2 | 2 | 2 | 0 | 1 | 0 | 25 | 75 |
| 1980-1993 | Skjemmeneset | 76.5656 | 16.5854 | 3 | 3 | 1 | 0 | 1 | 0 | 15 | 85 |
| 1980-1993 | Tokrossoya | 76.5600 | 16.2989 | 1 | 3 | 2 | 1 | 1 | 0 | 85 | 15 |
| 1980-1993 | Langstranda | 76.6297 | 16.3169 | 1 | 2 | 3 | 1 | 1 | 1 | 75 | 25 |
| 1980-1993 | Breineset | 76.8006 | 15.7847 | 1 | 2 | 3 | 1 | 0 | 0 | 15 | 0 |
| 1980-1993 | Palffyodden | 76.8985 | 15.5192 | 1 | 2 | 3 | 1 | 0 | 0 | 15 | 0 |
| 1980-1993 | Lisbetelva | 76.9192 | 15.6767 | 1 | 3 | 3 | 1 | 0 | 1 | 15 | 0 |
| 1980-1993 | Hofferpynten | 76.9458 | 15.2570 | 1 | 3 | 2 | 1 | 0 | 1 | 10 | 90 |
| 1980-1993 | Stone City | 76.9622 | 15.9393 | 1 | 2 | 2 | 1 | 0 | 1 | 0 | 15 |
| 1980-1993 | Samarinvagen | 76.9667 | 16.1902 | 3 | 1 | 1 | 1 | 0 | 1 | 0 | 15 |
| 1980-1993 | Bauten | 76.7211 | 16.1562 | 3 | 1 | 1 | 1 | 0 | 1 | 0 | 15 |
| 1980-1993 | Selbukta | 77.0100 | 16.2700 | 3 | 1 | 1 | 1 | 0 | 1 | 0 | 15 |
| 1980-1993 | Hyrneodden | 77.0267 | 16.0578 | 2 | 2 | 1 | 1 | 0 | 0 | 0 | 15 |
| 1980-1993 | Luciapynten | 77.0500 | 15.9100 | 2 | 2 | 1 | 1 | 0 | 1 | 0 | 15 |
| 1980-1993 | Gnalodden | 77.0152 | 15.8784 | 2 | 1 | 1 | 1 | 0 | 1 | 0 | 15 |
| 1980-1993 | Isbjornhamna | 77.0058 | 15.5981 | 1 | 2 | 2 | 1 | 0 | 1 | 10 | 90 |
| 1980-1993 | Rotjespynten | 77.0058 | 15.3172 | 1 | 2 | 3 | 1 | 0 | 0 | 90 | 10 |
| 1980-1993 | Hyttevika | 77.0503 | 15.1431 | 1 | 3 | 3 | 1 | 0 | 0 | 15 | 0 |
| 1980-1993 | Nottinghambukta | 77.0650 | 15.1081 | 1 | 3 | 3 | 1 | 0 | 1 | 15 | 0 |
| 1980-1993 | Vimsodden | 77.1117 | 15.0294 | 1 | 1 | 3 | 1 | 0 | 1 | 15 | 0 |
| 1980-1993 | Kapp Borthen | 77.1714 | 14.4489 | 1 | 2 | 3 | 1 | 0 | 0 | 15 | 0 |
| 1980-1993 | Steinodden | 77.2844 | 14.2639 | 1 | 2 | 3 | 1 | 0 | 0 | 15 | 0 |
| 1980-1993 | Dunderbukta | 77.4789 | 13.9953 | 1 | 2 | 3 | 1 | 0 | 0 | 50 | 50 |
| 1980-1993 | Klokkeodden | 77.5583 | 14.0764 | 1 | 3 | 3 | 1 | 0 | 0 | 15 | 0 |
| 1980-1993 | Calypsobyen | 77.5594 | 14.5178 | 1 | 3 | 3 | 1 | 0 | 0 | 75 | 25 |
| 1980-1993 | Vestervagen | 77.5167 | 14.5239 | 2 | 1 | 1 | 1 | 0 | 1 | 0 | 15 |
| 1980-1993 | Fagerbukta | 77.5056 | 14.7669 | 2 | 1 | 1 | 1 | 0 | 1 | 25 | 75 |
| 1980-1993 | Malbukta | 77.5503 | 14.9253 | 1 | 3 | 2 | 1 | 0 | 0 | 0 | 15 |
| 1980-1993 | Ingebritsenbukta | 77.5439 | 15.0989 | 3 | 2 | 2 | 0 | 0 | 0 | 0 | 15 |
| 1980-1993 | Slettebu | 77.5353 | 15.3422 | 3 | 2 | 1 | 0 | 0 | 0 | 0 | 15 |
| 1980-1993 | Sore Nathorstmorenna | 77.8561 | 16.0794 | 3 | 1 | 1 | 0 | 0 | 1 | 0 | 15 |
| 1980-1993 | Mullerneset | 78.4878 | 12.3542 | 2 | 2 | 2 | 1 | 0 | 0 | 20 | 80 |
| 1980-1993 | Kaffioyra | 78.6294 | 11.9425 | 2 | 2 | 2 | 1 | 0 | 1 | 10 | 90 |
| 1980-1993 | Ossian Sarsfjellet | 78.9669 | 12.4611 | 3 | 1 | 1 | 1 | 0 | 1 | 0 | 15 |
| 1980-1993 | Kapp Guissez | 79.0689 | 11.6597 | 2 | 2 | 2 | 1 | 0 | 0 | 10 | 90 |
| 1980-1993 | Ministerodden | 79.2661 | 12.0381 | 3 | 1 | 1 | 0 | 0 | 1 | 0 | 15 |
| 1980-1993 | Smeerenburg | 79.6239 | 11.4033 | 1 | 2 | 2 | 1 | 0 | 0 | 10 | 90 |
| 1980-1993 | Idrottnesset | 79.8300 | 12.1958 | 1 | 2 | 3 | 1 | 0 | 0 | 0 | 15 |
| 1980-1993 | Einsteinodden | 79.0431 | 16.2894 | 3 | 2 | 1 | 0 | 0 | 1 | 0 | 15 |
| 1980-1993 | Dirksodden | 79.6906 | 15.6858 | 2 | 2 | 2 | 1 | 0 | 0 | 0 | 15 |
| 1980-1993 | Bangenhuk | 79.8739 | 15.7003 | 1 | 2 | 3 | 1 | 0 | 0 | 10 | 90 |
| 1980-1993 | Verlegenhuken | 80.0583 | 16.2383 | 1 | 1 | 3 | 1 | 0 | 0 | 5 | 90 |
| 1980-1993 | Eremitten | 79.1672 | 19.2497 | 3 | 1 | 2 | 0 | 1 | 1 | 0 | 15 |
| 1980-1993 | Tumlingodden | 78.9687 | 20.4034 | 3 | 1 | 2 | 0 | 1 | 1 | 0 | 15 |
| 1980-1993 | Lewinodden. Isfjorden | 78.0798 | 13.7203 | 1 | 3 | 3 | 1 | 0 | 1 | 15 | 0 |
| 1980-1993 | Gronfjorden | 78.0968 | 13.9655 | 1 | 1 | 2 | 1 | 0 | 0 | 80 | 20 |
| 1980-1993 | IsfjordRadio | 78.0609 | 13.6256 | 1 | 3 | 3 | 1 | 0 | 0 | 50 | 50 |
| 1980-1993 | Colesbukta | 78.1097 | 15.0197 | 2 | 1 | 2 | 0 | 0 | 1 | 10 | 90 |
| 1980-1993 | Longyearbyen | 78.2253 | 15.6328 | 1 | 1 | 1 | 0 | 0 | 1 | 0 | 30 |
| 1980-1993 | Grumant | 78.1753 | 15.1053 | 1 | 2 | 2 | 0 | 0 | 0 | 0 | 30 |
| 1980-1993 | Piramiden | 78.6544 | 16.3658 | 3 | 1 | 1 | 0 | 0 | 1 | 0 | 30 |
| 1980-1993 | Skotehytta | 78.6997 | 16.6078 | 2 | 2 | 1 | 0 | 0 | 1 | 0 | 30 |
| 1980-1993 | Bohemanflya | 78.3820 | 14.6610 | 2 | 3 | 2 | 1 | 0 | 1 | 0 | 30 |
| 1980-1993 | Erdmanpynten | 78.2483 | 14.0882 | 2 | 2 | 2 | 1 | 0 | 0 | 80 | 20 |
| 1980-1993 | Tryghamna | 78.2217 | 13.8342 | 2 | 2 | 1 | 1 | 0 | 1 | 50 | 50 |
| 1980-1993 | Diabasodden | 78.3628 | 16.1353 | 2 | 2 | 2 | 0 | 0 | 0 | 0 | 20 |
| 1980-1993 | Vindodden | 78.3350 | 16.5669 | 2 | 2 | 2 | 0 | 0 | 1 | 0 | 20 |
| 2000-2016 | Wilczekodden | 77.0000 | 15.5300 | 1 | 2 | 2 | 1 | 0 | 0 | 31 | 0 |
| 2000-2016 | Agskjera. Isfjorden | 78.2022 | 12.9901 | 1 | 2 | 2 | 1 | 0 | 0 | 42 | 0 |
| 2000-2016 | Hamburgbukta | 79.5262 | 10.6939 | 1 | 3 | 1 | 1 | 0 | 0 | 71 | 0 |
| 2000-2016 | Calypsobyen. Bellsund | 77.5468 | 14.5630 | 1 | 1 | 2 | 1 | 0 | 1 | 8 | 52 |
| 2000-2016 | Steinneset. Bellsund | 77.7538 | 14.4392 | 1 | 2 | 2 | 1 | 0 | 1 | 46 | 2 |
| 2000-2016 | Lewinodden. Isfjorden | 78.0798 | 13.7203 | 1 | 3 | 3 | 1 | 0 | 0 | 55 | 5 |
| 2000-2016 | Ny Alesund | 78.9277 | 11.9312 | 1 | 1 | 2 | 1 | 0 | 1 | 38 | 30 |
| 2000-2016 | Erdmanpynten | 78.2483 | 14.0882 | 1 | 2 | 2 | 1 | 0 | 0 | 80 | 20 |
| 2000-2016 | Bohemanflya | 78.3820 | 14.6610 | 1 | 3 | 2 | 1 | 0 | 0 | 30 | 0 |
| 2000-2016 | Piramiden | 78.6530 | 16.3647 | 1 | 2 | 1 | 1 | 0 | 0 | 0 | 60 |
| 2000-2016 | Gronfjorden | 78.0968 | 13.9655 | 1 | 2 | 2 | 1 | 0 | 0 | 80 | 0 |
| 2000-2016 | Linneelva | 78.0797 | 13.7408 | 1 | 1 | 2 | 1 | 0 | 0 | 500 | 3 |
| 2000-2016 | inneelva W | 78.0797 | 13.7194 | 1 | 2 | 2 | 1 | 0 | 1 | 10 | 0 |
| 2000-2016 | Isfjordradio W | 78.0558 | 13.5808 | 1 | 2 | 3 | 1 | 0 | 0 | 10 | 0 |
| 2000-2016 | Randvika | 78.0719 | 13.6756 | 1 | 1 | 2 | 1 | 0 | 0 | 10 | 0 |
| 2000-2016 | Tryghamna S | 78.2070 | 13.8145 | 1 | 3 | 1 | 1 | 0 | 0 | 10 | 0 |
| 2000-2016 | Tryghamna N | 78.2501 | 13.7478 | 1 | 3 | 1 | 1 | 0 | 0 | 10 | 0 |
| 2000-2016 | Ymerbukta S | 78.2529 | 13.9250 | 1 | 1 | 1 | 1 | 0 | 1 | 10 | 0 |
| 2000-2016 | Gronfjord NW | 78.0752 | 14.0391 | 1 | 2 | 1 | 1 | 0 | 0 | 10 | 0 |
| 2000-2016 | Linneelva E | 78.0837 | 13.7521 | 1 | 1 | 3 | 1 | 0 | 1 | 10 | 0 |
| 2000-2016 | Gronfjord SW | 77.9994 | 14.1819 | 1 | 2 | 2 | 1 | 0 | 0 | 10 | 0 |
| 2000-2016 | Gronfjord S | 77.9642 | 14.2544 | 1 | 2 | 1 | 1 | 0 | 0 | 10 | 0 |
| 2000-2016 | Gronfjord E | 78.0035 | 14.2911 | 1 | 2 | 3 | 1 | 0 | 0 | 10 | 0 |
| 2000-2016 | IsfjordRadio SW | 78.0331 | 13.6105 | 1 | 3 | 3 | 1 | 0 | 0 | 10 | 0 |
| 2000-2016 | IsfjordRadio SW1 | 78.0534 | 13.5763 | 1 | 3 | 3 | 1 | 0 | 0 | 10 | 0 |
| 2000-2016 | IsfjordRadio | 78.0609 | 13.6256 | 1 | 3 | 3 | 1 | 0 | 0 | 10 | 0 |
| 2000-2016 | Longyearbyen. | 78.2253 | 15.6328 | 1 | 1 | 1 | 1 | 0 | 1 | 0 | 30 |
| 2000-2016 | Fuglehuken Fyrr (FF) | 78.8964 | 10.4667 | 1 | 2 | 3 | 1 | 0 | 0 | 7 | 4 |
| 2000-2016 | E.Coast 0.5 km from FF | 78.8987 | 10.5056 | 1 | 2 | 3 | 1 | 0 | 0 | 10 | 28 |
| 2000-2016 | Carmichelpynten | 78.8894 | 10.5720 | 1 | 2 | 3 | 1 | 0 | 0 | 29 | 8 |
| 2000-2016 | E.Coast 3km from FF | 78.8868 | 10.6608 | 1 | 2 | 2 | 1 | 0 | 0 | 38 | 5 |
| 2000-2016 | E.coast 4.5 km from FF | 78.8818 | 10.7355 | 1 | 2 | 2 | 1 | 0 | 0 | 20 | 0 |
| 2000-2016 | E. Coast 6km. Aberdeenflya | 78.8754 | 10.7889 | 1 | 2 | 2 | 1 | 0 | 0 | 24 | 0 |
| 2000-2016 | W Coast 400m from Lighthouse | 78.8897 | 10.4682 | 1 | 2 | 3 | 1 | 0 | 0 | 6 | 6 |
| 2000-2016 | Fuglehuken Fyrr | 78.8972 | 10.4652 | 1 | 2 | 3 | 1 | 0 | 0 | 9 | 2 |
| 2000-2016 | E.Coast 2 km from FF | 78.8894 | 10.5720 | 1 | 2 | 3 | 1 | 0 | 0 | 18 | 2 |
| 2000-2016 | Ymerbukta | 78.3019 | 13.9481 | 1 | 2 | 2 | 1 | 0 | 1 | 1 | 35 |
| 2000-2016 | Ny Alesund harbor | 78.9300 | 11.8723 | 1 | 1 | 2 | 1 | 0 | 0 | 9 | 29 |
| 2000-2016 | Ny Alesund beach | 78.9286 | 11.9341 | 1 | 1 | 2 | 1 | 0 | 0 | 15 | 10 |
| 2000-2016 | Fuglefjelet - Isfjorden | 78.1961 | 15.1844 | 1 | 1 | 2 | 1 | 0 | 0 | 3 | 28 |
| 2000-2016 | Bohemannesset Isfjorden | 78.3819 | 14.7533 | 1 | 2 | 2 | 1 | 0 | 0 | 3 | 0 |
| 2000-2016 | Russekjella | 78.0800 | 13.7200 | 1 | 2 | 2 | 1 | 0 | 0 | 265 | 0 |
| 2000-2016 | Kapp Linne | 78.0600 | 13.5800 | 1 | 1 | 3 | 1 | 0 | 0 | 40 | 0 |
| 2000-2016 | Bohemanflya | 78.3800 | 14.6600 | 1 | 2 | 2 | 1 | 0 | 0 | 69 | 0 |
| 2000-2016 | W Bohemannest | 78.3800 | 14.6600 | 1 | 2 | 2 | 1 | 0 | 0 | 675 | 0 |
| 2000-2016 | Brimerpyten | 78.5500 | 16.2600 | 1 | 2 | 2 | 1 | 0 | 0 | 0 | 29 |
| 2000-2016 | Festningodden | 78.1000 | 13.9700 | 1 | 2 | 3 | 1 | 0 | 0 | 42 | 0 |
| 2000-2016 | Russekjella | 78.0800 | 13.7400 | 1 | 2 | 2 | 1 | 0 | 0 | 23 | 0 |
